# Effects of Protein Supplementation on Performance and Recovery in Resistance and Endurance Training

**DOI:** 10.3389/fnut.2018.00083

**Published:** 2018-09-11

**Authors:** Harry P. Cintineo, Michelle A. Arent, Jose Antonio, Shawn M. Arent

**Affiliations:** ^1^Center for Health and Human Performance, Rutgers University, New Brunswick, NJ, United States; ^2^Department of Health and Human Performance, Nova Southeastern University, Davie, FL, United States; ^3^Department of Kinesiology and Health, Rutgers University, New Brunswick, NJ, United States

**Keywords:** protein, athlete, endurance, strength, nutrient timing

## Abstract

There is robust evidence which shows that consuming protein pre- and/or post-workout induces a significant rise in muscle protein synthesis. It should be noted, however, that total daily caloric and protein intake over the long term play the most crucial dietary roles in facilitating adaptations to exercise. However, once these factors are accounted for, it appears that peri-exercise protein intake, particularly in the post-training period, plays a potentially useful role in terms of optimizing physical performance and positively influencing the subsequent recovery processes for both resistance training and endurance exercise. Factors that affect the utility of pre- or post-workout feeding include but are not necessarily limited to: training status (e.g., novice vs. advanced, or recreational vs. competitive athlete), duration of exercise, the number of training sessions per day, the number of competitive events per day, etc. From a purely pragmatic standpoint, consuming protein post-workout represents an opportunity to feed; this in turn contributes to one's total daily energy and protein intake. Furthermore, despite recent suggestions that one does not “need” to consume protein during the immediate (1 h or less) post-training time frame, it should be emphasized that consuming nothing offers no advantage and perhaps even a disadvantage. Thus, based on performance and recovery effects, it appears that the prudent approach would be to have athletes consume protein post-training and post-competition.

## Introduction

Dietary protein plays a critical role in countless physiological processes in the body. The current Recommended Dietary Allowance (RDA) for healthy individuals is 0.8 g/kg/day (1). It is increasingly evident, however, that protein intake of at least 1.4–1.6 g/kg/day (2) would be more appropriate for active individuals attempting to optimize training adaptations. In an effort to meet this threshold, protein supplements are often consumed. In 2015, protein powder sales were valued at 4.7 billion U.S. dollars and were second only to sport drinks in the sports nutrition market (3). The popularity of protein supplements is likely influenced by the claims of increased muscle mass, increased fat loss, improved performance, and improved markers of recovery.

To date several meta-analyses, reviews, and systematic reviews have attempted to quantify and clarify these claims, but with mixed results (2, 4–7). However, these efforts are complicated by the fact that the populations studied included trained and untrained, healthy normal weight, overweight or obese individuals, as well as injured, movement impaired, and those with metabolic or other diseases states. Additionally, the emphasis of recent reviews has been largely on impacts on muscle protein synthesis (MPS), hypertrophy, and body composition, with most of the outcomes pertaining solely to resistance training (2, 4–7). Performance and recovery effects have been given secondary consideration at best, and these are areas that would be of particular interest to most athletes or athletic individuals.

Furthermore, performance and recovery outcomes, as well as physiological adaptations, are unique to the modality of training primarily employed. Anaerobic training refers to short bouts of high intensity movements which are often interspersed with longer recovery periods between efforts, with two of the most popular applications being resistance training or interval training (8). On the other hand, aerobic or endurance training refers to exercise bouts that primarily rely on oxidative phosphorylation and can last from minutes to hours (9). This latter type of training has received almost no consideration in recent protein reviews. Whether engaging in resistance or endurance training, protein supplementation may have the potential to enhance or complement exercise-induced physiological responses. The purpose of this review is to examine these potential performance and recovery applications of protein supplementation for both resistance and endurance training, with emphasis placed on studies utilizing various “peri-exercise” supplementation protocols within ~60 min pre- or post-training in healthy, exercising individuals.

## Protein supplementation and resistance training

A recent comprehensive review by Jager et al. (2) identified a number of key issues related to protein intake in healthy, exercising individuals. Of particular note, the importance of protein intake during and around a training session for recovery and performance appears to be dependent on total daily protein intake, as well as presence or absence of an energy deficit. While findings do support the effect of post-exercise protein intake on increases in fat free mass (FFM), individuals consuming adequate daily calories and a minimum daily protein intake of 1.6 g/kg may not see any added benefit of immediate post-training protein consumption on muscular strength (2). However, Morton et al. (7) suggested that the strength (and hypertrophy) effects of additional post-resistance training protein supplementation may be greater in those with previous resistance training experience and that the magnitude of this effect is somewhat mitigated with aging. Furthermore, it is important to note that resistance-trained individuals in a caloric deficit require significantly more protein to offset any potential loss of lean body mass, with optimal daily protein intake for these individuals potentially being in the range of 2.3–3.1 g/kg FFM (10). While this recommendation increases total caloric intake from protein, resulting in the necessity to decrease energy intake from fat and carbohydrate, protein appears to have unique characteristics, and overfeeding with protein has been shown to have no negative effects on body composition in trained individuals (11). Similarly, healthy, older adults also require a greater quantity of total daily protein (0.61 g/kg FFM) compared to their younger counterparts (0.25 g/kg FFM) (12). Additionally, as a percentage of total daily energy intake, older adults must increase the contribution from protein due to decreases in energy intake, as well as protein's ability to attenuate sarcopenia by increasing muscle hypertrophy, subsequently maintaining or increasing muscular strength and power (13).

It has previously been demonstrated that ingestion of milk-based protein following a damaging eccentric resistance protocol helps to attenuate the expected decrements in strength and repeated sprint ability from 24 to 72 h following the bout (14–16). Recently, a group of researchers found that whey protein can facilitate muscle recovery following an intense isotonic exercise bout as well and that it is more than just an issue of caloric replacement (17). They compared the effects of a whey protein supplement (25 g protein, 2.5 g fat, and 3 g CHO) to a calorie-equated carbohydrate drink (32.5 g CHO) in resistance-trained young men performing an acute, total body resistance training protocol, and assessed performance variables at 10- and 24-h post-exercise. A moderate beneficial effect on acute anaerobic power and strength was found in the group that consumed the protein supplement, suggesting that there may have been improvements in rate of recovery over those who consumed the carbohydrate drink (17). This is particularly notable given that the subjects were already habitually consuming 1.9 g/kg/d of protein and may hold particular relevance for athletes engaging in high-intensity, explosive sports.

It has been suggested that protein quality may have an effect on both acute and chronic adaptations to exercise (2, 18, 19). Protein quality is a measure of a given protein source's ability to provide adequate quantities of the essential amino acids required for protein synthesis (20). Additionally, leucine, a branched-chain amino acid (BCAA), has been shown to be a prerequisite stimulator of skeletal MPS, which is critical for both the recovery and adaptive processes following a training bout (21). Given some of the favorable outcomes seen with ingestion of certain complete proteins, particularly milk-based and, more specifically, whey proteins (2), questions have been raised about the possible application of other protein sources that may be lower in leucine content. Two recent investigations have studied the effects of the quality of a post-exercise protein source on performance and recovery (22, 23). Each of these studies took a unique approach to determining the differences in physiological changes following exercise and protein supplementation. Fabre et al. (22) compared the effects of 20 g of whey protein, 10 g of whey protein plus 10 g of casein protein, and 4 g of whey protein plus 16 g of casein protein consumed post-exercise in 31 recreationally resistance-trained males. Following 9 weeks of resistance training 4 days per week, no differences in changes in body composition, muscular strength, or muscular endurance were found, suggesting all three protein supplements were equally effective. When comparing 16 g of beef protein, 18 g of whey protein, and a calorie-equated carbohydrate drink consumed post-resistance training 3 days per week for 8 weeks in 42 recreationally resistance-trained males, no differences in changes in body composition, muscle thickness, or performance variables were found (23). One limitation of each of these studies is that they failed to control for total daily energy and macronutrient intake; therefore, subjects may have already been consuming adequate total daily calories and protein so the additional protein, regardless of its source, failed to result in any additional improvements in performance or body composition.

While most protein supplement resistance training studies have used a “post-exercise” administration protocol, it is possible that timing effects extend to the entire peri-workout period. Schoenfeld et al. (24) examined the effects of consuming 25 g of hydrolyzed whey protein immediately prior to a resistance training session with a 3-h fast post-exercise vs. consuming the same quantity and source of protein immediately following the same training session after having fasted for 3 h in 21 resistance-trained males. All subjects were consuming a 500-kcal surplus and 1.8 g/kg of protein daily. No differences in changes in body composition or one-rep max back squat or bench press were found between the groups after the 8-week intervention. Along with the findings from other studies (22, 23, 25), these data support the idea that protein intake post-workout may not be critical as long as protein is consumed prior to training or total daily protein intake is adequate. However, this does not preclude the possibility that pre- and post-exercise supplementation would be even more beneficial depending on dose.

To interpret the disparate effects of protein supplementation on resistance training performance, a few issues should be taken into account. The training stimulus must be adequate to result in strength improvement, regardless of protein timing, total protein intake, or nutritional status. Protein supplementation by individuals participating in ineffective resistance training programs will be less impactful. The beginning training status of individuals also appears to play a significant a role in any potential benefit seen as a result of protein consumption on strength, hypertrophy, and body composition (7). While the main focus of this paper is the healthy, trained individual, it is worth noting that protein supplementation for novice individuals may not confer any additional benefit above and beyond that of the training intervention (5). However, as training status increases, so does the potential effect of protein supplementation for improving performance and recovery.

Alternatively, Reidy and Rasmussen (6) have proposed the existence of a “protein paradox” wherein well-trained individuals may require less dietary protein due to the increased efficiency of protein turnover in this population. However, it should be noted that this is speculative and has not been fully substantiated by the available research, particularly for performance-related outcomes. Even taking this into account, one factor that appears to be just as important as total daily protein intake in well-trained individuals is the utilization of a specific protein dosing strategy based on body weight or FFM. Additionally, Thomson et al. (12) showed that healthy, older adults may also benefit from a higher protein intake in addition to a protein dosing strategy to adequately stimulate MPS. Thus, the appropriate timing or pacing of protein intake throughout the day may optimize results from resistance training (26). While recent critical or meta-analytic reviews have argued that protein timing is inconsequential after accounting for total protein intake (6, 27), there are two factors that must be taken into account when considering these conclusions. First, very few “timing” studies have actually been conducted. In most cases, the studies were not designed to compare time of administration, but rather type or quantity of nutrient (or placebo) ingested post-exercise. Second, only a few of the included studies used trained subjects. Most employed novice exercisers. One of the studies that has found a benefit of protein timing (28) was conducted in experienced resistance-trained males. Again, this may lend credence to the notion that training status matters when considering protein supplementation strategies. Additionally, it should be noted that strength improvements not reaching statistical significance may prove to be significant in areas of individual competition or performance. Very few studies have actually utilized highly trained individuals or athletes, so translating the current findings to this population should be done with caution. Finally, it is worth noting that several studies have shown the addition of carbohydrate and creatine monohydrate to a protein supplement, typically whey protein, results in greater strength and hypertrophy improvements from resistance training programs (26). Though a detailed discussion of these other macronutrients is beyond the scope of this review, these results do point to an overall “nutrient” impact as well as possible synergistic effects.

Perhaps a driving factor in performance (i.e., strength or power) improvements with peri-workout protein supplementation could be enhanced recovery, which would potentially translate to enhanced capacity for an increased training load stimulus. Recovery from exercise has been measured through many different methods in previous research. Delayed onset muscle soreness (DOMS), which is defined as an aching pain in a given muscle following a novel exercise bout, has been measured subjectively (29). Though the cause of DOMS is multifaceted and tied to a cascade of events linked to muscle damage, it is not necessarily an indicator of the magnitude of muscle damage and, therefore, cannot be used by itself to determine muscular recovery and adaptations from exercise (29). Specific biomarkers and MPS rates appear to be the most efficient and widely used methods of objectively determining muscle breakdown, recovery, and adaptation from exercise. Acute elevations of cortisol and creatine kinase (CK) are two biological indicators of muscle damage and the subsequent recovery processes that can be measured through blood sample analysis (30, 31). Post-exercise muscle biopsies can be used to determine rates of MPS, which directly measure the magnitude of the recovery process immediately following exercise (32).

West et al. (17) measured recovery variables following a total-body resistance training session and found that those subjects who consumed a whey protein supplement (25 g protein, 2.5 g fat, and 3 g CHO) had lower rates of whole body protein breakdown, while those who consumed a carbohydrate supplement (32.5 g CHO) actually had higher rates of whole body protein synthesis. The protein group, however, appeared to improve whole body net protein balance over 24 h post-exercise. As noted previously, the subjects were already consuming 1.9 g/kg/d protein, so additional protein through supplementation may have been less impactful. Interestingly, there was no difference between total body net protein balance between the groups. It should be noted that whole body protein synthesis is not necessarily a reflection of skeletal muscle protein synthesis (33). Kim et al. (33) discovered that net protein (whole body) balance was superior with a 70 vs. 40 g dose consumed prior to a resistance-training protocol. However, no differences were found in muscle protein synthesis between the 40 and 70 g dose. Thus, one must not conflate measures of whole body protein metabolism with those of skeletal muscle.

Nevertheless, the recovery of muscle function has been demonstrated in other studies (15, 16) of milk protein supplementation after eccentric exercise, perhaps due to myofibrillar protein remodeling. The results of these studies further support the idea that protein consumed post-exercise is crucial for maximizing rates of protein synthesis in skeletal muscle. The effect on total body protein balance, however, is still a bit unclear. Carbohydrates have been shown to have a protein sparing effect, therefore the combination of protein and carbohydrate to decrease rates of muscle protein breakdown (MPB) and increase rates of MPS may be the best strategy for shifting total body protein balance to the net anabolic side (34), even if carbohydrate itself does not necessarily enhance MPS (35, 36). This may partially explain the benefits of the milk supplement used by Cockburn et al. (15) and Cockburn and Stevenson (16) as it also contained carbohydrate. Perhaps there is a synergistic effect.

In addition to the investigations discussed earlier regarding post-exercise protein quality and training adaptations, Burd et al. (25) also measured markers of recovery through protein synthesis. The researchers collected muscle biopsies and measured rates of MPS following resistance training. In the 0–2 h post-exercise window, the group that consumed 30 g of protein in the form of skim milk expressed higher rates of MPS than the group that consumed 30 g of protein from beef (25). However, rates of MPS in the 2–5 h post-exercise window did not differ. This may be explained by the rate of digestion and absorption of these protein sources. Protein from dairy, specifically the whey portion, appears to be absorbed faster, and elicit a faster MPS response than beef.

The difference between whole egg and protein-equated egg white consumption post-exercise was also studied recently (37). The researchers measured rates of MPS through muscle biopsies and found that the group that consumed the whole egg exhibited higher rates of MPS. One limitation to this study was the lack of control for total calories and macronutrients. The whole egg treatment consisted of 18 g of protein, 17 g of fat, and 223 kcal, while the egg white treatment consistent of 18 g of protein, 0 g of fat, and only 73 kcal (37). While the discrepancy in calories between treatment groups may have impacted total daily calories, thus impacting MPS, one cannot ignore the possibility of the role that differences in macronutrients may play.

Lastly, a 2017 investigation looked at the differences between protein-equated native whey protein, whey protein concentrate, and milk (38). Native whey protein is produced through the filtration of raw milk, while whey protein concentrate is a byproduct of cheese production. Native whey protein consists of undenatured proteins and has a higher leucine content (38). Each treatment consisted of ~20 g of protein, ~6 g of fat, and ~40 g of carbohydrates but contained 2.7, 2.2, and 2.0 g of leucine, respectively. The supplements were ingested immediately after and again 2 h post-exercise following a moderate intensity lower body resistance training session. Results showed higher blood amino acids concentrations in native whey and whey protein concentrate than in milk. MPS was elevated in the whey protein condition from 1 to 3 h post, while it was elevated 1–5 h post in the native whey condition. There was no difference in MPS 1–5-h post-workout between native whey and whey protein concentrate, though MPS was higher from 1 to 5 h post-workout in the native whey condition compared to milk (38). Collectively, these data support that whey protein, regardless of its levels of processing (i.e., native whey protein vs. whey protein concentrate), increase MPS by similar magnitudes that are greater than those of milk alone. How this translates to long-term differences remains to be determined.

## Protein supplementation and endurance training

While the majority of the literature regarding the effects of protein intake on performance has focused on anaerobic activities, more recent work has examined its role on endurance activities, but this has mostly been absent from the most recent reviews. Similar to resistance training, the impact appears to be at least somewhat dependent on the presence or absence of other nutrients, particularly carbohydrate. A 2010 systematic review and meta-analysis compared 11 studies investigating the effects of consumption of protein and carbohydrate vs. consumption of carbohydrate alone during a bout of cycling on performance during a subsequent bout of cycling (39). Across the 11 studies, consumption of protein and carbohydrate resulted in an average improvement of 9% in performance (defined as time to exhaustion and time trial performance) compared to consumption of carbohydrate alone (39). To investigate if the increased caloric intake due to inclusion of protein was responsible for this improved performance, a further analysis of isocarbohydrate and isocaloric conditions was performed. Examination of isocarbohydrate conditions yielded a 10.5% improvement in overall performance, while isocaloric conditions resulted in 3.4% improvement (39), suggesting that the improvements due to protein inclusion were not simply due to increased calories. When considering only those studies measuring performance by time trial (3), improvements were not statistically significant. However, studies utilizing time to exhaustion protocols (8) did result in statistically significant improvements. It is worth noting that in all studies showing statistically significant improvement, whey protein was the source of protein utilized, though differences between concentrate vs. isolate were not quantified. Again, it is prudent to highlight that performance improvements not reaching statistical significance may have clinical or practical relevance, specifically for athletes. For example, a 1% improvement in performance would have been the difference in winning the Gold Medal instead of the Silver Medal in the men's marathon in the 2016 Olympic Games in Rio. Therefore, even seemingly “trivial” differences do indeed have a significant effect on performance and outcomes at the elite level.

When discussing the impact of protein on performance, it is imperative to include the impact that protein may have on glycogen replenishment and subsequent exercise performance. Standard discussions of glycogen replenishment focus solely on carbohydrate consumption. Recommendations for adequate post-exercise carbohydrate consumption are to consume 0.6–1.0 g/kg carbohydrate within 30 min of cessation of exercise and again every 2 h for the next 4–6 h (40, 41). Carbohydrate consumption of 1.2 g/kg every 30 min over 3.5 h also resulted in maximal glycogen replenishment (41, 42). In cases of suboptimal post-exercise carbohydrate consumption, the addition of protein can improve glycogen replenishment and decrease symptoms of muscle damage (43). Practical applications of standard post-exercise carbohydrate consumption recommendations may be limited in real world situations. Moreover, athletes training multiple times daily may have fewer opportunities to consume recovery meals or have an elevated need for “rapid” recovery, including rehydration, to facilitate the subsequent training session. Beyond just glycogen replenishment aspects, it has also been shown that the presence of protein in rehydration beverages can enhance intestinal fluid uptake, aiding in rehydration (44) and that BCAA consumption during endurance exercise may improve time trial performance and peak power output while improving markers of immune health (45) and attenuate serotonin levels, subsequently resulting in a delay of central fatigue (46).

A systematic review by Pasiakos et al. (5) investigated the relationship between protein, muscle function, and recovery. The authors included studies that measured markers of muscle damage followed by a test of physical performance or muscle function. Populations of the review included healthy individuals with daily dietary protein intake at or above the current RDA of 0.8 g/kg per day. While some of the endurance exercise studies included showed decreases in markers of muscle damage, such as CK, or decreases in muscle soreness in groups consuming protein after initial exercise bout (47–49), many did not (50–52). This may have resulted from the inclusion of studies utilizing both trained and untrained subjects, as well as individuals consuming suboptimal daily protein intakes. Despite the reduced plasma CK levels and muscle soreness, consumption of protein did not result in improvements in subsequent performance measures when repeat performance was tested < 24 h following the initial bout. This evidence suggests that plasma CK levels, perceived level of muscle soreness, and muscle function may only be modestly related or perhaps utilizing a single method of measure paints an inadequate picture of recovery due to individual variability (5). Without additional studies to clarify these relationships, developing guidelines based on these markers as representing recovery may be ill-advised. Individuals must be cautious when attempting to measure recovery from exercise based on these metrics alone. For example, a recent study of 20 high-level soccer players tested the effects of a milk protein concentrate supplement (80% casein and 20% whey) compared to an isocaloric carbohydrate-containing placebo on high intensity running performance, knee extensor and flexor strength, and antioxidative capacity over the course of a 1-week in-season microcycle consisting of two games separated by 2 days (53). On game days (days 1 and 4), the supplement was consumed immediately post-, 3 h post-, and 6 h post-match in three different doses of 25, 30, and 25 g, respectively, resulting in a total of 80 g. On training days (days 2, 3, 5, and 6), 20 g of the supplement was consumed with breakfast. High intensity running performance, measured as distance covered at speeds >19 km/h, was greater during the last 15 min of game two following protein supplementation. Additionally, knee extensor concentric strength recovered quicker after the first game following protein supplementation. Endogenous antioxidant concentrations were greater following game two only in the protein-supplemented condition. Though soccer is a “power-endurance” sport rather than simply an endurance sport, these findings hold relevance for understanding the impact of protein intake on recovery and repeated performance in actual athletes.

Since 2014, additional work investigating the impact of protein consumption on biochemical markers of metabolic status, physiological fatigue, and recovery in endurance-trained athletes has been performed (54). For 5 weeks, elite or experienced marathon runners received either 33.5 g/day of whey protein or maltodextrin 30 min following the completion of each training session leading up to a race covering marathon distance. Blood samples were collected to assess biochemical markers of metabolism, muscle damage, and fatigue and took place prior to beginning the intervention, 1 day following the marathon, and 1 week following the marathon. These markers included CK, lactate dehydrogenase (LDH), AST, and ALT. Runners who supplemented with whey protein displayed decreased AST and ALT compared to maltodextrin-supplemented runners. CK and LDH, biochemical indicators of muscle damage, were significantly greater in the maltodextrin group post-marathon compared to the whey protein-supplemented group. Elevations in CK and LDH were still significant 1-week post-marathon in the maltodextrin group compared to the whey protein group (54). The whey protein group also showed significantly decreased triglycerides (TG) and total cholesterol (TC) compared to the maltodextrin group post-marathon. The maltodextrin group actually showed increased TC levels. Only the whey protein group showed significant decreases in LDL post-marathon and at 1 week post-marathon (54). The authors suggested that the decrease in TC seen in whey-supplemented runners may indicate that cholesterol was more efficiently converted to steroid hormones, resulting in improved physiological recovery and adaptations from the strenuous exercise bout. One week post-marathon, most biomarkers of damage and stress were still significantly lower in the whey protein group compared to the maltodextrin group (54). In addition to the more favorable biomarker profiles in the protein supplemented group, performance in the 12-min run/walk test was also greater in the whey protein-supplemented group 1-week post-marathon. Together, these results indicate that whey protein supplementation during marathon preparation and recovery, and that the supplement aids in attenuating metabolic and muscular damage. Daily dietary assessments were not included in this study (54), thus limiting possible practical applications or recommendations. As we have addressed previously, caloric deficit or daily protein consumption <1.4–1.6 g/kg may potentiate the effect of peri-workout protein consumption on recovery and subsequent performance. Further studies are necessary to elucidate the potential contribution of peri-workout whey protein ingestion on makers of muscle damage, recovery, and subsequent performance measures in endurance athletes.

In real-world sport performance situations, recovery and performance must be evaluated in the context of an accumulated effect. The ability to train consistently while remaining healthy is critical for continued progression and optimal performance. Endurance athletes in particular are at increased risk for upper respiratory tract infections (55). Factors contributing to this increased risk may include reduced immune function through low circulation of certain T-lymphocytes, especially during periods of increased volume and/or intensity of training. A diet providing a daily protein intake of 3 g/kg, including 60 g/day of casein protein, has been shown to be sufficient in returning circulating immune cell levels to those seen during lighter training periods, while a diet providing a daily protein intake 1.5 g/kg did not result in enhanced immune cell levels (56, 57). Kephart et al. (45) have also found this beneficial effect on the immune system to extend to BCAA supplementation in doses of 12 g/d in trained cyclists.

Additionally, Rowlands et al. (58) found that consumption of ~64 g protein over 3 h following intense endurance exercise resulted in gene expression favorable for improving substrate, specifically fatty acid, mobilization and mitochondrial proteins for oxidation, especially in the electron transport chain. Post-exercise consumption of protein at levels thought to maximally stimulate MPS would potentially not have this same impact. Post-exercise protein consumption affects other systems and pathways and should not be considered only in terms of stimulating MPS. As further evidence of this notion, Levenhagen et al. (59) demonstrated that 10 g of casein protein enhanced MPS following 60 min of moderate intensity endurance exercise. Although this supplementation protocol stimulated MPS, subjects were found to be in negative whole-body protein balance. Because prolonged bouts of endurance exercise (i.e., >2 h) result in considerable oxidation of amino acids, specifically leucine, and intense or prolonged bouts of endurance exercise result in hypoxia-mediated small intestinal injury, negative whole-body protein balance may be common in endurance athletes (60–62). Because of this, protein requirements and recommendations for endurance athletes must consider more than MPS, especially since short-term increases in MPS do not fully explain the dynamics of long-term whole-body net protein balance and various training adaptations.

## Conclusions and future direction

Overall, total daily energy and protein intake over the long term play the most crucial dietary roles in facilitating adaptations to exercise. However, once these factors are accounted for, it appears that peri-exercise protein intake plays a potentially useful role in optimizing physical performance and positively influencing the subsequent recovery processes. Challenges surround the definition of “performance” and the appropriate metrics by which to measure it based on desired outcomes. Difficulties also arise in attempting to define and quantify the concept of recovery. Additionally, both performance and recovery must be viewed in context depending on whether the emphasis is an immediate, short-term effect (i.e., 24 h or less) or a long-term training response.

It should also be noted that protein timing, whether it is pre-, during, or post-workout, is often framed within the context of bodybuilding (i.e., the singular goal of increasing skeletal muscle mass). It is evident that to use such a narrow frame of reference ignores the potential utility of protein timing within the context of endurance events (i.e., running, cycling, rowing, swimming, triathlon, etc.), as well as the vast majority of individual and team sports in which skeletal muscle hypertrophy is not a pre-eminent concern. For instance, if one competes in a weight-class sport (e.g., boxing, mixed martial arts, weightlifting, powerlifting, etc.), gains in body weight or lean body mass are often avoided; otherwise, the individual athlete would need to compete in a heavier weight class. In these situations, protein timing in particular may serve a useful role in recovery.

Translating research into practical application requires differentiation between novice or trained individuals, healthy normal weight or healthy overweight individuals, special populations, or those with certain metabolic or disease states. Here, we specifically focus on healthy, exercising individuals and limit our conclusions to these individuals. It is important moving forward that the study populations used are appropriate for the goals of the study and desired applications. For example, it is of little use to have a sample of recreationally-trained individuals if the goal is to understand performance in high-level athletes.

Though protein-containing meals result in increase of MPS on their own, as does resistance training, the timing of ingestion of protein around exercise further enhances this increase of MPS (63, 64). It is worth noting that an upper limit for this acute dosing has not really been established, though there is evidence that 40 g of protein stimulates MPS to a greater degree than 20 g following whole-body resistance training (65). A dose higher than this, however, has not been included using the same timing paradigm. In reality, the “ideal” amount of peri-exercise protein consumption depends on many factors, including total caloric intake, total daily protein intake, training status of the individual, age of the individual, FFM, type of protein consumed, type and amount of other nutrients consumed, and the composition and timing of the most recent pre-training meal.

Much attention has been given to daily protein consumption and thresholds that must be met for peri-training protein consumption to exert additional benefit (>1.6–2.2 g/kg/d). As such, pre-, intra-, and post-training nutrient consumption present additional opportunities for athletes to contribute to their daily protein intake total and can be viewed in the context of ways to meet these “larger” daily needs by optimizing intake.

With regard to endurance exercise, protein consumption during exercise may not confer an immediate ergogenic benefit, especially when carbohydrate consumption is adequate. It may, however, aid in delaying central fatigue, reducing MPB, and contributing to a more positive, whole-body nitrogen balance. Additionally, protein consumption in and around intense or prolonged endurance activity may aid in reduction of upper respiratory tract infection incidence and improved immune system function. It may also aid in upregulating gene expression of proteins necessary for improving bioenergetic pathways. The impact of this on subsequent training sessions should not be dismissed and is an important part of improving performance.

The effect of protein consumption on resistance training is highly dependent on many variables not related to protein. The combination of peri-training protein consumption with inadequate or ineffective resistance training protocols will not maximize improvements in strength or hypertrophy. Resistance training protocol interventions must be of adequate intensity, volume, and frequency with an emphasis on progressive overload to produce results. Additionally, adequate training interventions coupled with calorie-restricted nutrition protocols may require increased protein intake of 2.3–3.1 g/kg FFM to yield desired improvements in strength, hypertrophy, or maintenance of FFM (10). Consideration must also be made for the age of resistance-trained individuals, as older adults require protein intake over and above that of their younger counterparts to receive the same benefits noted above (66).

In order to fully understand the role of protein (or any substrate for that matter) on performance, the practical application beyond the contrived training or recovery interventions presented must be addressed. Daily training schedules of athletes require an ongoing ability to recover and perform. As an example, most of the studies included in this area utilized a training protocol that took ~3–4 h per week, typically in moderately-trained individuals. For comparative purposes, a competitive athlete may spend 3–10 times this amount of time training per week (if not more). For this reason, the “window” for recovery should be considered to encompass each and every hour between training and competition. Protein dosing strategies need to take this into account. This becomes even more apparent when considering that the uniform distribution of protein throughout the day results in greater MPS than an uneven distribution even when total daily protein intake is equal (67). Arciero et al. (64) demonstrated the combination of resistance training and consumption of 4–6 meals per day containing 20–40 g of protein per meal resulted in positive changes in body composition and physical performance. These results suggest that the pattern of daily protein ingestion may also impact results from resistance training protocols and provides further evidence that we must look beyond the few hours following training to determine the impact that protein may have on performance and recovery. Further evidence in support of extending the “recovery window” concept are results from nighttime protein ingestion studies. Madzima et al. (68) found that consumption of 30 g of casein, 30 g of whey, or 33 g of carbohydrate 30 min prior to sleep resulted in increased resting energy expenditure and improved VO_2_ the following morning. While no statistically significant changes were observed between groups, protein groups trended toward greater increases when compared to the carbohydrate group while morning fat oxidation was greatest in the casein supplemented group.

Taken together, these data demonstrate the need for a more comprehensive view and methods of measuring recovery. Increased sensitization of muscle to protein and nutrients for 24–72 h following training coupled with multiple weekly training sessions results in an on-going state of recovery. Because of this, we need to begin considering this longer stimulus window as an opportunity to maximize feeding, rather than as a reason why immediate post-workout ingestion may not be particularly important. In other words, consuming nothing post-workout would be an unwise strategy if the goal is to potentially optimize the adaptive response to exercise training.

Overall, there appears to be no adaptive advantage to avoiding protein intake in the peri-workout period. Stimulation of MPS in the acute period following training may not result in improvements in strength, hypertrophy, body composition, or performance without deliberate implementation of additional strategies during the prolonged recovery period. As such, this much broader view should be considered with regard to future investigations.

## Author contributions

JA and SA conceived the topic. HC, MA, JA, and SA wrote the paper.

### Conflict of interest statement

SA is on the Advisory Panel for Dymatize. JA is the CEO of the International Society of Sports Nutrition—an academic non-profit that receives grants in part from companies that sell dietary protein.

The remaining authors declare that the research was conducted in the absence of any commercial or financial relationships that could be construed as a potential conflict of interest.

The reviewer CK declared a past co-authorship with several of the authors SA and JA to the handling Editor.
